# Monitoring Study in Honeybee Colonies Stressed by the Invasive Hornet *Vespa velutina*

**DOI:** 10.3390/vetsci9040183

**Published:** 2022-04-12

**Authors:** Ana Diéguez-Antón, María Shantal Rodríguez-Flores, Olga Escuredo, María Carmen Seijo

**Affiliations:** Department of Vegetal Biology and Soil Sciences, Faculty of Sciences, University of Vigo, 32004 Ourense, Spain; ana.dieguez.anton@uvigo.es (A.D.-A.); mariasharodriguez@uvigo.es (M.S.R.-F.); oescuredo@uvigo.es (O.E.)

**Keywords:** *Vespa velutina*, honeybee colonies, weather conditions, monitoring systems

## Abstract

*Vespa velutina* is an invasive species that is currently the main concern for beekeeping in some areas of northern Spain. The hornet hunts honeybees to feed its larvae, stressing and weakening the honeybee colonies. To avoid losses of honeybee colonies, it is essential to investigate the pressure that is exerted by the yellow-legged hornet on apiaries and its consequences. In the present study, hives were monitored in an apiary that was situated in a high-pressure area of *V. velutina* during the years 2020 and 2021. The monitoring of environmental conditions of the apiary, the internal conditions of the colonies, and a hunting camera were used to relate the presence of hornets in front of the hives to the weather conditions in the apiary and the consequences caused on the colonies. The relationships between weather conditions and the hornet’s activity showed two types of hornet behavior. In the months of July and August, the maximum number of hornets appeared in non-central hours of the day. Meanwhile, in the months of September and October, the highest pressure in the apiary occurred in the central hours of the day, coinciding with temperatures between 15 °C and 25 °C and a relative humidity that was higher than 60%. The honeybee colony with the highest thermoregulatory capacity was the strongest and it was the key factor for the colony survival even when the hornet pressure was high too. Therefore, strengthening the hives and improving beehive health status is essential to avoid colonies decline.

## 1. Introduction

The invasive hornet *Vespa velutina nigrithorax* du Buysson, 1905 (Hymenoptera: Vespidae) in Europe is known for its spectacular expansion and its ability to adapt to new environments resulting in a notable threat to the endemic fauna and biodiversity [1]. For this reason, it has been included by European Union in the black-list of invasive alien species (Regulation EU 1141/2016) for which it is mandatory to develop surveillance plans and actions to limit its spread as well as control and containment strategies [2].

*V. velutina* was introduced along two routes from southern China, establishing viable and expanding populations in northeast Asia (South Korea and Japan) [3,4] and Europe (France) [5]. In Europe, the first detection was in 2004, and later in 2010 they were detected in North Spain [6,7], colonizing the Cantabric and Atlantic coast by the end of 2012. In this area, the favorable climatic conditions, and the ability of the species to adapt to different environments has allowed the species to reach a very high reproductive success rate [8,9]. Nowadays, more than 20,000 nests each year in Galicia (Northwest Spain) are recorded [10].

The yellow-legged hornet is a social insect living in colonies that can reach more than 13,000 individuals annually [11]. The life cycle is sensitive to weather conditions starting at the end of winter (normally February), in the most suitable places. The queen emerges from its winter shelter and builds the first nest (embryo nest), starting in a few days to lay eggs which originate the first workers. After that, the population increases either over this nest or in a new nest (secondary nest) [11]. The first nests can commonly be found in buildings of urban and semiurban areas and the secondary ones in tree branches, but also in the ground, human constructions, or abandoned beehives [8]. The emerged queen uses sugar sources such as nectar or fruit juices as food, but *V. velutina* brood needs animal protein to grow. To get these proteins, hornet workers hunt, becoming a generalist predator that attacks a wide range of insects and spiders [12,13]. Particularly, honeybees constitute one of the most preferable diets, with other studies mentioning that more than half of the prey that are captured are honeybees and social wasps [12].

The apiaries are an attractive source of food because there is a high concentration of honeybees. The hornets hunt foraging honeybees when they return to their colonies by hovering in front of the hive entrance, grabbing the honeybees in flight, and killing them. Then, the honeybee thorax is selected, due to the high protein content that is provided by the flight muscles and it is transported to the hornet’s nest to feed the larvae [2]. The impact of *V. velutina* on honeybee colonies is not well quantified but in high colonized areas of Galicia, beekeepers’ associations registered the collapse of all the hives of the apiary due to the pressure of this invasive species (unpublished data). In France it was reported that 30% of the hives were destroyed/weakened in Gironde in 2010 due to *V. velutina* [14], while in the southwest of France, beekeepers reported losses of between 30% and 80% of honeybee colonies each year, resulting in poor production of honey and other beehive products [2]. In addition to the honeybee mortality, the colonies suffer a prolonged period of confinement because the hornet pressure at the entrance limits the activity of the foraging bees outside the hive. This period of lockdown causes stress, having a severe impact on the colony strength with consequences in both the short- and long-term [15,16]. In addition, the honeybees cannot forage, which means that they cannot collect enough food to maintain themselves and consume what they have stored in the hive. In our geographical area, prolonged periods of confinement have been observed starting in mid-June and extending into the winter. Usually, the bee colonies weaken and are unable to survive the pressure [17].

Galicia has a census of 193,192 hives [18], and despite only 6% of the beekeepers being professionals (having more than 150 hives), apiculture is a complementary economic activity that is important for maintaining the population in rural areas. Each year, the presence of *V. velutina* in apiaries causes significant damages in beehives. Consequently, the beekeepers face significant economic investments searching for control alternatives to reduce the hornets’ pressure and to replace the dead colonies. The most common control methods that are used are based on several types of baits to trap the hornets, entrance reducers, hives muzzle, rackets, and electric harps [2,19,20,21,22,23,24,25]. However, the impact of the yellow-legged hornet continues to be relevant, varying according to the territory and environmental conditions. Predation pressure changes throughout the day, but it is not well understood what factors affect this behavior and whether this pressure affects the internal conditions of the honeybee hives. In this work, beehive monitoring systems and recording systems were used for monitoring an apiary. The main objective was to find out how the predation behavior of *V. velutina* varies on the beehives regarding environmental conditions and the influence of this species on honeybee colonies depending on their strength.

## 2. Materials and Methods

### 2.1. Study Area and Study Period

The apiary was situated in a hornet’s high-pressure area (Galicia, NW Spain; 42°49′ N 8°27′ W) where *V. velutina* nests had been first reported in 2015 [10]. This area represents an Atlantic climate that is characterized by subdued temperatures, abundant rainfall, mild winters and summers, and low annual thermal oscillation [8]. The most abundant melliferous plants in the area are *Castanea sativa*, *Eucalyptus globulus*, and shrubs as *Rubus* and heathers (*Erica*).

The apiary faced southwest at an elevation of 260 m and included a total of ten conventionally Langstroth beehives that were managed by an experienced beekeeper. It was the experimental apiary of Agroforestry Training and Experimentation Centre of Sergude, (Boqueixón, Spain). The study period covered the years 2020 and 2021, starting in February 2020 and ending in October 2021. There were two colonies of *Apis mellifera* that were monitored during this period.

### 2.2. Beehive Monitoring System

The Bee Hive Monitoring System from Slovakia [26] was used to monitor the beehives and meteorological conditions of the apiary. The system allowed the recording and storage of data from the different sensors and the periodic transfer of data to a remote server for subsequent data analysis. All the data were collected in a GSM (Global System for Mobile) gateway that allowed sensor data to reach the user without the need to visit the apiary because it worked with a SIM phone card. The system allowed registering external and internal conditions of beehives. The parameters that were recorded for the external conditions were temperature (°C) and relative humidity (%). The internal sensors provided the temperature and relative humidity of the colony. The internal sensors were placed inside the beehive between the inner cover and the center of brood chamber. The data was recorded every 10 min. From each sensor, 94,000 data points were analyzed.

### 2.3. Photo Monitoring System

In addition to the data sensors, an external camera that was located in front of the beehives was used (APEMAN 20MP 1080P Hunting Camera 940 nm IR Brightness LED Free Night Detection, IP66 Waterproof). The camera was placed at a distance (around 1 m) that made it possible to distinguish *V. velutina* and the honeybees in the photographs in addition to the visualization of the studied hives. The area of the photo window was 200 × 150 cm² and sightings were counted when the hornets were in front of each colony. The camera was programmed to take photographs every hour, from 6:00 h to 0:00 h. Finally, 9567 photographs were obtained and analyzed.

### 2.4. Management Practices and Beehives Surveillance

The management practices that were carried out in the apiary during the study period were implemented by the beekeeper according to the needs of the colonies. The monitoring systems were installed in the hives in February 2020 when the study started. During spring and summer suppers were added to the hives when needed. Honey harvest took place in September in both years (2020 and 2021). After that, chemical treatments to control *Varroa* mites were performed. Finally, in October and November, the colonies were fed with a sugar solution in water (1:1) 1 kg of this solution per week and per hive if needed. Bait traps and electric harps were used to reduce the hornets’ pressure in the apiary in the summer and autumn months. Moreover, the conditions of the study were field conditions, allowing the beekeeper to manage the apiary as he usually did.

The strength of the colonies for each year was measured according to the modified protocol that was proposed by Delaplane et al. [27]. First the colonies were opened and the frames were removed and photographed on each side. The combs were photographed with the bees but if they had a large number of bees that did not allow observation of brood, honey, or pollen, then the bees were brushed, and the comb was photographed again. The digital photos were analyzed using the computer program ImageJ. The results were calculated in percentage, being the total size of the frame 100%. The same procedure was repeated twice per year in each colony.

### 2.5. Statistical Analysis

The data were processed using IBM SPSS Statistics^®^ for Windows version 22.0 (IBM Corp., New York, NY, USA, 2013). A Spearman non-parametric correlation analysis was performed to assess the relationship between the hornets’ sightings and the meteorological parameters (external temperature, external relative humidity, and precipitation). The level of significance was calculated for the 99% (*α* < 0.01) confidence interval. The related variables were the meteorological conditions and the hornets’ sightings in the hornets’ pressure period (from June to October of both years). In addition, a comparative analysis was carried out between the honeybee colonies by month for each year. For this, an independent samples *t*-Test was used (*α* < 0.05).

## 3. Results

### 3.1. Environmental Conditions and V. velutina Sightings

Of the two years that were studied, 2020 was hotter and drier than the monitored period in 2021. In 2020, the mean external temperature, relative humidity, and precipitation were 13.5 °C, 92.4%, and 4.1 L/m^2^ compared to 12.9 °C, 91.9%, and 5.5 L/m^2^ in 2021, respectively. The mean external temperature in May, July, and October showed significant differences between both years (*p* < 0.05). The mean external temperature of May and July 2020 were higher than 2021, while the value that was obtained for October 2021 was higher than the value of October 2020. About the external relative humidity, May, July, and September of 2021 had higher values than those months in the 2020 year, nevertheless, the external relative humidity of October was higher in 2020. The precipitation values were higher from May to October 2021 than in 2020, except in August that had lower precipitation in 2021.

**Figure 1:**
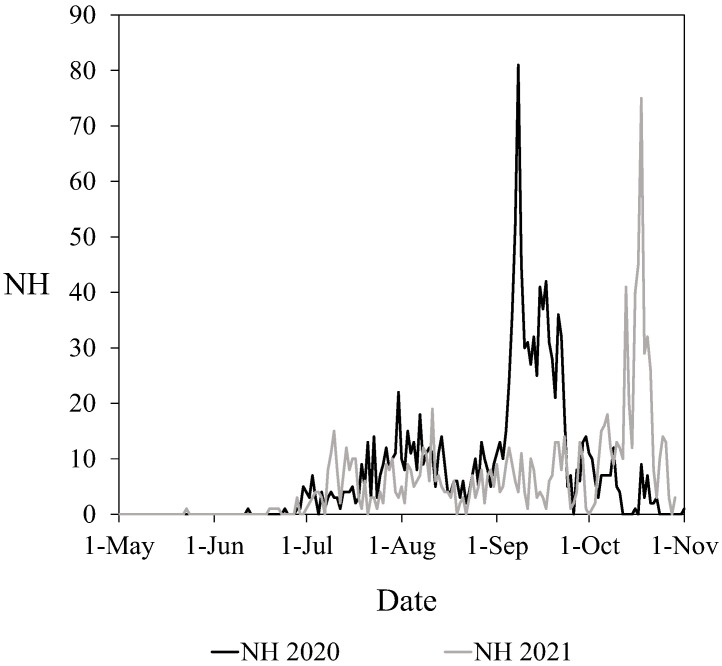
The total number of hornet sightings (NH) per day counted in the apiary by year (2020 and 2021).

The first *V. velutina* sightings that were captured by the camera in the apiary were found on 12 June 2020, and 26 May 2021, with very few specimens (Figure 1). In both years, the beekeeper noticed the presence of the hornets in the apiary in July, one month later than the camera showed. Therefore, the camera allowed notification of the appearance of *V. velutina* before the beekeeper’s warnings. In the beginning, the hornets appeared sporadically and in many days, there were no observations. From June to mid-August, the number of hornets in front of the hives increased continuously. After that, the number of hornets decreased in both years and then increased exponentially again until mid-September in 2020 and mid-October in 2021. In 2020, after the maximum number of hornets in September, the curve decreased. In the 2021 period, the curve was similar to 2020 but its peak was shifted by about one and a half months with respect to the peak that was observed in 2020.

**Figure 2:**
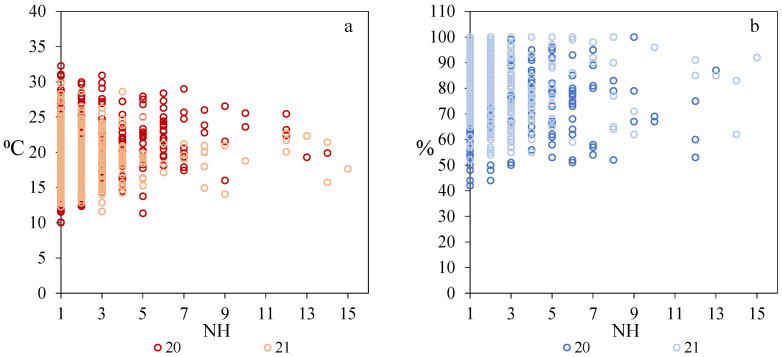
Relationship between the number of hornets (NH) that were found in front of the hives and the external parameters in both years, 2020 and 2021. (**a**): temperature (°C), (**b**): relative humidity (%).

The external temperature and relative humidity were related to the presence of hornets in the apiary. The hornets appeared when the temperature values were between 5 °C and 33 °C and the values of relative humidity between 40% and 100% (Figure 2). The highest number of hornets was found with temperatures between 15 °C and 25 °C and a high relative humidity (>60%).

Correlations were performed to understand the relationship between the hornet pressure and the meteorological parameters in the period when hornets were present in the apiary. There was a positive correlation between hornet sightings and the external temperature (*r* = 0.368, *p* < 0.01). Whereas inverse correlations were displayed between the hornets’ sighting and the external relative humidity (*r* = −0.347, *p* < 0.01). As the pattern of hornets’ pressure was different each month; the correlations were also different depending on the month that was studied. The months of July and August had a lower correlation between the external temperature and *V. velutina* sightings as a result of the bimodal distribution. Whereas, in June, September, and October a higher correlation was observed between the external temperature and the number of hornets in front of the hives.

### 3.2. Daily Pattern of V. velutina Occurrence

**Figure 3:**
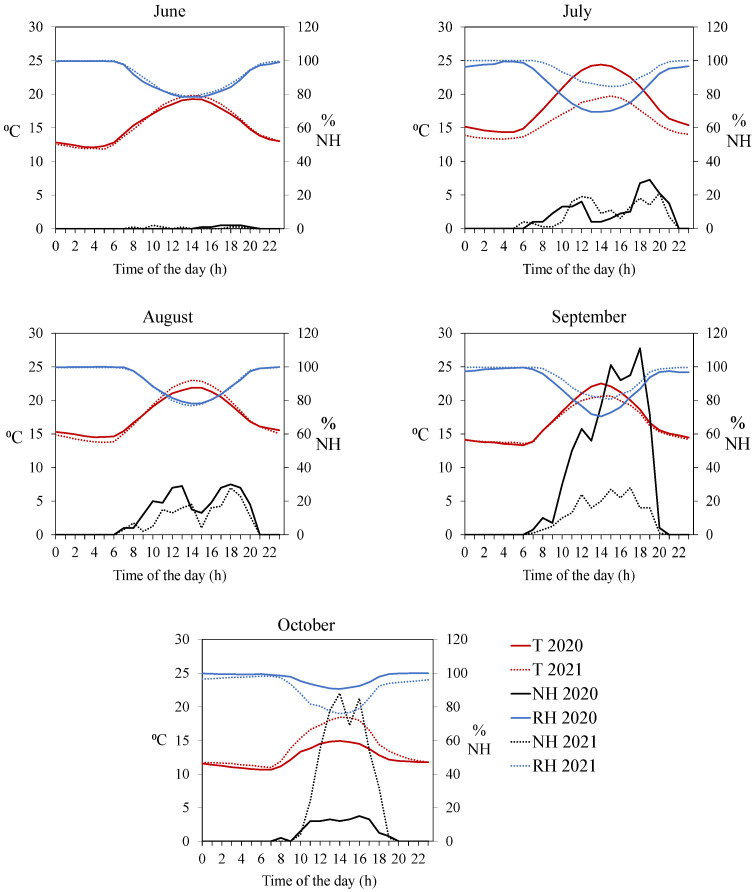
*V. velutina* sightings (NH), external temperature (T), and external relative humidity (RH) in the months with the highest pressure of hornets in front of the colonies: June, July, August, September, and October of both years (2020 and 2021).

The presence of hornets varied during the daytime and depending on the months and the year of study. Figure 3 shows the hourly mean external temperature, the hourly mean relative humidity, and the accumulated sum of hornets around the beehives by month in both years. The hourly distribution of the number of hornets that were counted made it possible to differentiate two periods in the two years sampled: the first from June to August and the second from September to October. From June to August, most of the hornets appeared usually in the non-central hours of the day. While in the months of September and October, the highest number of hornets was found in the mid-day.

For both years, the first sightings in the apiary were found in the month of June. Into the summer, during the months of July and August, the highest number of *V. velutina* individuals were concentrated in the apiary in the non-central hours of the day 10:00–13:00 and 16:00–20:00 in both years (Figure 3). Hornets avoided maximum temperatures and minimum relative humidity between 13:00 h and 16:00 h. Thus, September 2020 was the month in which the maximum number of hornets appeared in the apiary. The period between 16:00 h and 19:00 h was characterized by presenting high temperatures (although not the maximum ones) and low relative humidity. In September 2021, for the external temperature, the pattern was similar. While in October 2021, the hornets increased exponentially in the central hours of the day, coinciding with the same weather conditions as in September 2020. In general, throughout the period, *V. velutina* began to appear at 7:00 h and finished at 21:00 h.

### 3.3. Colonies Strength and Hornets Pressure

Both colonies started the study with similar strength but promptly in March 2020 Colony 1 (C1) swarmed with a consequent decrease in the honeybee population and strength. Then, it began to weaken showing less brood, bee population, and food reserves than Colony 2 (C2), which presented higher strength. Both colonies progressed over the honey season and in winter reached the same status regarding brood (C1: 9%, C2: 14%), bee population (C1: 50%, C2: 60%), and food reserves (C1: 23%, C2: 19%). In spring 2021, there were no significant differences between the strength of the two colonies (brood C1: 35%, C2: 33%; food reserves C1: 36%, C2: 39%). However, during summer, C2 lost population and finally, at the end of the study, died.

**Figure 4:**
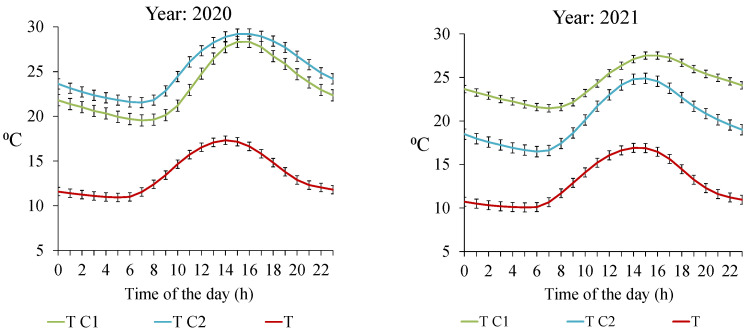
Mean and standard deviation values of the hourly external temperature (T) and internal temperature of the two colonies (T C1 and T C2) in the two years of study (2020 and 2021).

The thermoregulatory capacity of both colonies was compared. Some differences between the internal temperature of both colonies and the external temperature values were observed (Figure 4). In 2020, the difference in the hourly values of the internal temperature was lower than in 2021. In the first year, both colonies were able to maintain a high regulation of internal temperature; even C2 had better thermoregulation capacity than C1 (Figure 4). However, in 2021 the internal temperatures differed significantly between the colonies. In 2021, C1 presented a high thermoregulatory capacity during the day. On the contrary, the internal temperature curve of C2 was more influenced by the external temperature, showing a similar curve. Consequently, C2 had a better thermoregulatory capacity in 2020 than in 2021, and in 2021, C1 was the strongest one with the best thermoregulatory capacity.

The photos showed a total number of 2523 sightings in front of the monitored hives (1337 in 2020 and 1186 in 2021). Hornets’ sightings differed significantly between the colonies along the two years of study. In 2020, the highest number of hornets were found in July, August, and September with 700 sightings in front of C1 compared with 637 hornets in front of C2 (Table 1). The number of hornets per month did not show significant differences between the two monitored colonies (*p* > 0.01). In 2021, the months of June, July, and August had a similar pattern to 2020, with the highest number of hornets in front of C1. However, in September the pressure was considerably lower for C1, but in October, the highest number of hornets was detected in front of C2, with 457 hornets. Consequently, the total number of *V. velutina* in the 2021 season was 772 and 414 in front of C2 and C1, respectively. Significant differences in the number of hornets between C1 and C2 in the months of July, August, and October were found (*α* < 0.01).

## 4. Discussion

This study is a first approach evaluating the relationships between hornets, weather conditions such as temperature and relative humidity, hive management, and colony strength in Galicia. The invasive species was adapted very well to the territory, particularly in the areas with lower altitudes [8] and is currently considered one of the most key factors for honeybee colony losses. The voracious predation diminishes the honeybee population and disrupts colony stability, especially on those apiaries that are already stressed due to other factors [28]. The main factors affecting hornets’ pressure in apiaries were those that were dependent on the hornet population in the area: the number and size of nests, weather conditions, honeybee colonies strength, beekeeping management practices, and the use of control measures [29,30,31].

In the surrounding area of the apiary, 42 nests were detected and destroyed from May 2020 to October 2021, but despite this work to destroy nests, in both years there was high pressure on the apiary. The first sighting of hornets in the apiary was slightly earlier in 2021 than in 2020, whereas predation pressure lasted for more than six months in both years, as occurred in other countries where *V. velutina* is an invasive species [29]. The results showed that the hornets began to prey on the honeybees at the end of May or the beginning of June, one month before the beekeeper perceived the presence of the predator. In the first month, only a few hornets in front of the hives were found. During July and August, there was a higher maintained pressure with some differences between days. Finally, for both years it is worth highlighting the significant decrease in mid-August, followed by a notable increase in the number of hornets. In 2020, this occurred in September, whereas in 2021 was in October. The first period coincided with the first stages of the development of *V. velutina* nests. At this moment, the nests are small and produce few worker hornets, furthermore, the needs of the colonies are lesser. The first nests were detected in the area by citizens at the beginning of April [10] and the first hornets appeared in hives 50–60 days after.

There is scarce available information about the length of the different stages of the life cycle for *V. velutina* in the area. But some authors for *V. velutina auraria* indicated in laboratory conditions, the egg stage lasts 9–15 days, the larval stage for 10–18 days, and the pupal stage 15–20 days, with the length of the cycle being 33–53 days [32]. This suggests the onset of predation in hives occurs with the first cohort workers and pressure increases over time with the development of successive cohorts [33]. Hereafter, the number and size of nests in the environment increases, and therefore also the pressure on the apiaries. Removal of nests can briefly reduce predation, as occurred in both years in the middle of August. However, the area had strong pressure and quickly a high number of hornets appeared in front of the hives again. In the same way as other invaded areas, the highest pressure was in autumn (September 2020 and October 2021) [29,33,34], but this is contrary to its native area, when the highest pressure is observed in mid-summer [35]. At this moment, the hornets’ workers are highly active, preying honeybees due to nests having the greatest size and the need for protein to feed larvae. During this period, many hornets have been observed either in flight or in front of the hive, causing the greatest effects in the honeybee population [36,37,38]. The last hornets were seen in the apiary on 25 December 2020, despite active nests being removed in January 2021. As such, the hornet life cycle explains the activity of the hornets in the apiaries.

Favorable environmental conditions in early spring advance the emergence of queens from hibernation, the establishment of first queen nests, and probably, the larval development [11,33,39]. It has been shown that the moderate climatic conditions of Galicia favor the presence of this invasive species [8]. Hence, since the appearance of this species, this region is the place where a greater number of *V. velutina* nests have been recorded. The study area is characterized by an Atlantic climate with elevations below 500 m and is strongly influenced by the proximity of the coast. It is characterized by moderate temperatures and high annual regional rainfall observed especially in the months of December and January.

At the same time, weather affects hornets’ flight, and consequently, pressure on hives during the season and during the day. The results showed the external temperature and relative humidity affected the number of hornets in front of the hive throughout the day but also precipitation could affect this presence too. Another meteorological factor as wind speed was mentioned as more relevant for hornet’s flights and activity than temperature [34] and hornets’ activities outside the nest are influenced by the ultraviolet B (UVB) radiation [31]. The highest number of hornets was recorded when the temperature was close to 20 °C. At external temperatures above 35 °C and below 10 °C, no hornets were observed in front of the hives. Similarly, the highest number of hornets was recorded with an average external relative humidity of 80%. Precipitation reduced the number of hornets in the apiaries. This favorable temperature and relative humidity threshold marked a daily pattern distribution of hornets that varies along the season. Similar results were found in France where most of the sightings took place between 07:00 and 20:00 [38]. There were two different dynamics that were observed in July/August and in September/October and it is the first time that it is described. In the first period, the pressure of the hornets followed a bimodal distribution that started early in the morning until midday, when it dropped to rise again at night. Thus, the number of hornets increased in parallel with those hours when the temperature and relative humidity were more suitable for them. In the second period, the pressure of the hornets followed a continuous distribution throughout the day with the highest number of hornets appearing at midday. Thus, the presence of *V. velutina* in apiaries varies according to the month and time of day, as well as meteorological conditions. In the hottest months (July and August), two high peaks were observed throughout the day (at the beginning and the end of the day). This is contrary to what happens in their native area, where the highest pressure is in the morning and at midday [33,40].

More studies are needed to understand the hornet behavior in front of the hives, as well as the effectiveness of honeybees that are captured by the hornets during the day. Our study is a field trial under real conditions managed by the beekeeper. The monitoring of the internal conditions and the inspections of the hives allowed us to obtain information on the state of the colonies and the population they had. One of the parameters that was observed was the ability to maintain the temperature inside the hive (thermoregulation). A lack of internal thermoregulation has been confirmed to be related to weak colonies [41,42,43,44,45,46]. The two colonies behaved differently in both years in terms of thermoregulatory capacity. In this sense, C2 had less variation in internal temperature than C1, so C2 was able to conserve the temperature that was generated better than C1 in 2020. On the other hand, in 2021 C1 had a greater capacity for thermoregulation than C2, ending the study with the death of this colony. It was also observed that the number of hornets in front of C2 was very high, during the period of high pressure in the summer months until October. Honeybees consumed more honey in the colony and neglect internal temperature maintenance. This produced lower internal temperature to obtain the metabolic energy that is necessary for thermoregulation and honeybees spent more time in this task, and this is reflected in its weakening [45,46]. The more hornets that are observed in the apiary, the more it will affect the strength of the colonies. It was found that when the entrance was full of bees, the hornets would hide under the hive waiting to hunt the foraging bees when they returned [33,34].

**Figure 5:**
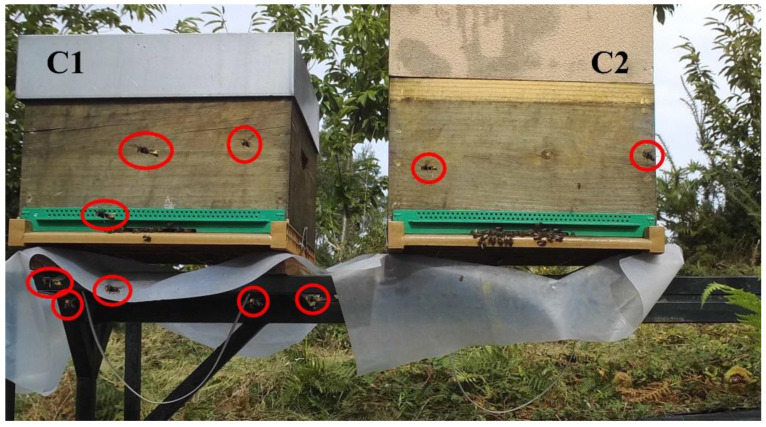
Hornets’ sightings marked with red circles in front of the two beehives. C1 on the left and C2 on the right. The picture was taken on 16 September 2020, at 16:00 h.

On the other hand, when there were fewer honeybees at the hive entrance, the hornets tried to enter directly. Several studies showed that hornet sightings in front of hive entrances are influenced by colony strength and health status [2,36,37]. Both colonies had different strengths during the study and according to this, C1 had more hornets in front of the hive than C2 in 2020 (Figure 5), while C2 had more hornets in front of the hive in 2021. It is known that hornets have a stationary flight, and they are moving between hives. However, weakened colonies showed less defense capacity and had fewer honeybees at the entrance so hornets use this gap to cause more pressure.

*A. mellifera* still exhibits an inefficient and disorganized defense against *V. velutina* [33,36,37], so it is important to control *V. velutina* and help colonies fight this threat. The use of some protection methods such as electric harps or muzzles can help reduce the pressure of the hornets. However, management techniques that are focused on strengthening colonies to facilitate defense behavior are essential. The influence of the distance between hives should also be evaluated to reduce the ability of hornets to catch honeybees when hives are close together. Hornets are looking for an opportunity to hunt so that the closer the hives are to each other, the more hunting options the hornet will have. Due to the intense pressure that is exerted during six months in the apiary, strong colonies can also become weak [36] and weak colonies with good management practices can survive and improve their health and strength. It is, therefore, very important to manage each honeybee colony individually according to its needs. Although C1 had a lower population and less vigor at the beginning of the study, with the management techniques that were applied, it arrived at the pre-wintering period with a high population of bees, new worker honeybees, and reserves for wintering. One of these management techniques was the use of electric harps between the hives. Despite the use of electric harps, this does not avoid the presence of hornets in front of the hives, although it has been proven that its use can be an effective tool to reduce pressure [25]. Another widespread practice is the use of the entrance reducer. When the population was too low, the reducer prevents the entrance of the biggest hornets in the colonies. However, when the population is high, it was observed a ball of bees at the entrance did not allow the foraging honeybees to land directly, so they stopped their flight at the entrance, making it easier to be hunted by hornets. This occurred with C2, which was initially stronger than C1, and became weak in the second year of study. These results showed that the use of the same management techniques for colonies with different strengths and health status may not be useful to avoid *V. velutina* consequences in apiaries. For this reason, *V. velutina* forces beekeepers to pay continuous attention to their apiaries and this is a major handicap for non-professional beekeeping.

In addition to honeybee losses, the presence of *V. velutina* in the apiaries affected the proper hibernation of the colony, which could lead to the weakening of the colonies for the next season. The stress *V. velutina* causes to honeybees occurs in a critical period for them because the collection of overwintering reserves by the honey bees and the increased number of hornets in the apiary hunting them occur simultaneously [16]. During the field visits, it was found that the hives had no food reserves and had to be fed continuously.

*V. velutina* preys mostly on honeybees [12,47], causing its population to decline, weakening the colonies until they die or become so weak that they cannot survive the winter [2,33,47]. There is not enough research studying how beekeeping management can reduce hornets’ consequences in apiaries and reduce beehives losses. Many of them are focused on implemented protection systems such as electric harps, muzzles, or bait traps, showing different efficacies [2,19,22,23,24,25]. However, the influence of the strength of the honeybee colony and the health status may play a decisive role in the survival of the honeybees for the next season because both pose a significant collapse risk on wintering honeybee colonies [24]. Therefore, *V. velutina* should be reported as an additional biological agent stressor in future studies of colony honeybee losses in affected areas.

## 5. Conclusions

This study is the first approach to *V. velutina* and *A. mellifera* colonies behavior in apiaries using monitoring systems. In the area of the study (Galicia, Spain), this invasive species is an important stressor for colonies, causing a predation pressure from June to the end of the year. The presence of hornets in the apiary is conditioned by their biological cycle, and the environmental conditions made the weak honeybee colonies more susceptible to collapse. As such, beekeeping management strategies have to be improved according to these factors.

## Figures and Tables

**Table 1:** Observations of hornets in front of each beehive per month and in each year.

	Colony	Jun.	Jul.	Aug.	Sep.	Oct.
NH 2020	C1	4	111	138	400	47
	C2	5	73	129	371	59
	*p*	0.772	0.241	0.832	0.839	0.563
NH 2021	C1	3	130	156	88	37
	C2	4	80	111	120	457
	*p*	0.730	0.004 *	0.001 *	0.390	0.008 *

C1: colony 1; C2: colony 2; NH: number of hornets corresponds to the total observed *V. velutina* individuals in the images. *: denotes significant differences between the colonies in the same month using an independent samples *t*-test (*α* < 0.01).

## Data Availability

The datasets that were generated during and/or analyzed during the current study are available from the corresponding author on reasonable request.
